# CAR immunotherapy in autoimmune diseases: promises and challenges

**DOI:** 10.3389/fimmu.2024.1461102

**Published:** 2024-10-01

**Authors:** Jingjing Yu, Yiming Yang, Zhanjing Gu, Min Shi, Antonio La Cava, Aijing Liu

**Affiliations:** ^1^ Hebei Medical University-National University of Ireland Galway Stem Cell Research Center, Hebei Medical University, Shijiazhuang, Hebei, China; ^2^ Department of Clinical Laboratory, The Second Hospital of Hebei Medical University, Shijiazhuang, Hebei, China; ^3^ Hebei Key Laboratory of Laboratory Medicine, The Second Hospital of Hebei Medical University, Shijiazhuang, Hebei, China; ^4^ Department of Medicine, University of California Los Angeles, Los Angeles, CA, United States; ^5^ Department of Medicina Molecolare e Biotecnologie Mediche, Federico II University, Naples, Italy; ^6^ Department of Rheumatology and Immunology, The Second Hospital of Hebei Medical University, Shijiazhuang, Hebei, China; ^7^ Hebei Research Center for Stem Cell Medical Translational Engineering, Shijiazhuang, Hebei, China

**Keywords:** CAR-T cells, autoimmune diseases, immunotherapy, double-negative T cells, γδ T cells, T regulatory cells, natural killer cells

## Abstract

In recent years, the use of chimeric antigen receptor (CAR)-T cells has emerged as a promising immunotherapy in multiple diseases. CAR-T cells are T cells genetically modified to express a surface receptor, known as CAR, for the targeting of cognate antigens on specific cells. The effectiveness of CAR-T cell therapy in hematologic malignancies including leukemia, myeloma, and non-Hodgkin’s lymphoma has led to consider its use as a potential avenue of treatment for autoimmune diseases. However, broadening the use of CAR-T cell therapy to a large spectrum of autoimmune conditions is challenging particularly because of the possible development of side effects including cytokine release syndrome and neurotoxicity. The design of CAR therapy that include additional immune cells such as double-negative T cells, γδ T cells, T regulatory cells and natural killer cells has shown promising results in preclinical studies and clinical trials in oncology, suggesting a similar potential utility in the treatment of autoimmune diseases. This review examines the mechanisms, efficacy, and safety of CAR approaches with a focus on their use in autoimmune diseases including systemic lupus erythematosus, Sjögren’s syndrome, systemic sclerosis, multiple sclerosis, myasthenia gravis, lupus nephritis and other autoimmune diseases. Advantages and disadvantages as compared to CAR-T cell therapy will also be discussed.

## Introduction

**Figure 1:**
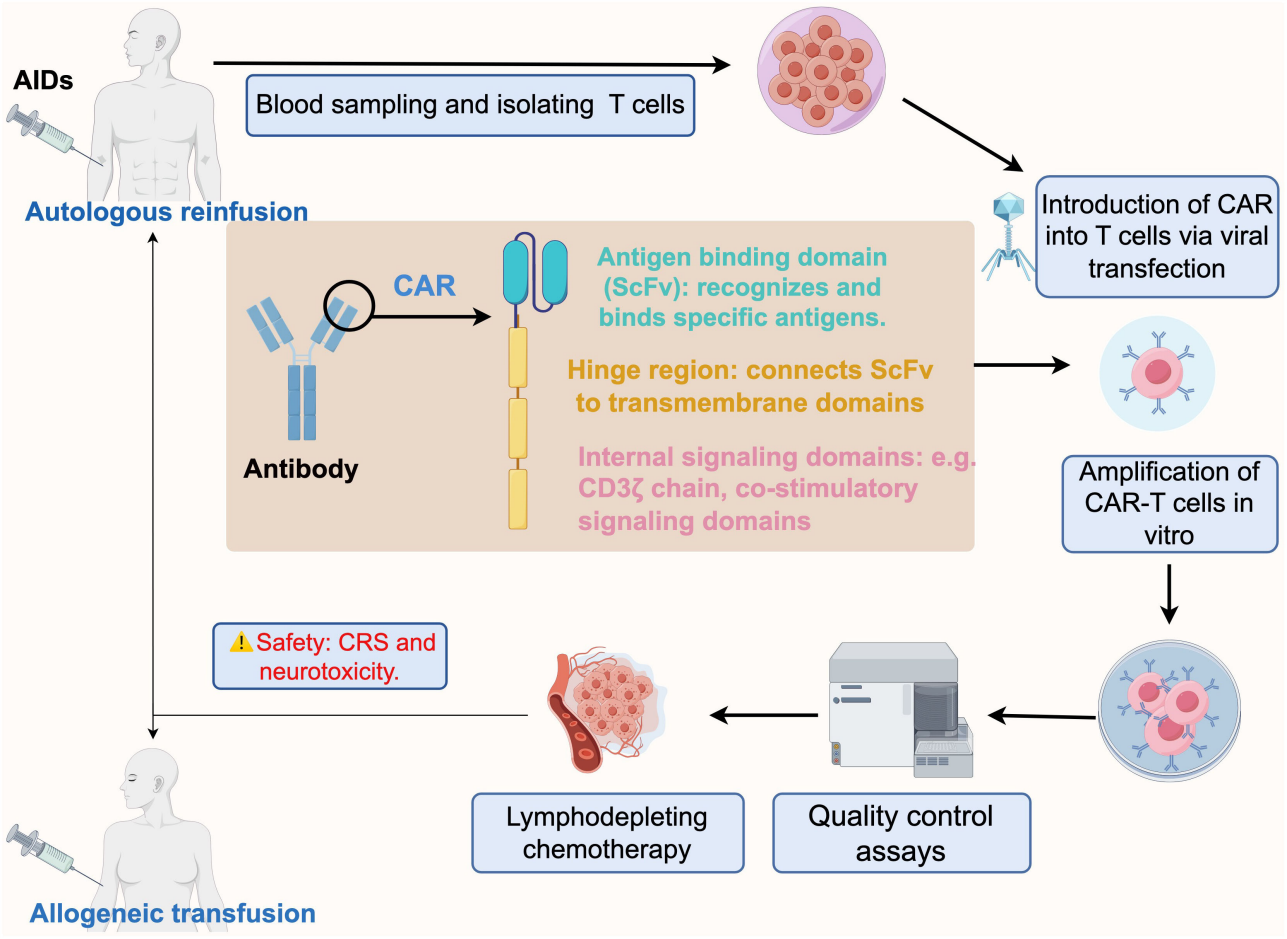
Schematic process of CAR-T immunotherapy for autoimmune diseases, from design to infusion. The CAR design initially incorporates a tumor antigen-specific ScFv that is fused to a major activation signaling component (usually CD3ζ). Blood is taken from patients with autoimmune diseases and T-cells are isolated, and the designed CAR is combined with T-cells via lentiviral transfection to expand CAR-T cells *in vitro*. The prepared CAR-T cells are subjected to several quality control assays, including sterility, cell activity, CAR expression level, cytotoxicity and specificity tests. Prior to CAR-T cell infusion, patients are usually required to undergo chemotherapy for lymphatic clearance to make space for incoming CAR-T cells. Finally, the qualified CAR-T cells are transfused back into the patient intravenously via autologous or allogeneic cell infusion. CAR-T Chimeric antigen receptor T cells, AIDs autoimmune diseases, ScFv single-chain variable fragment, CRS cytokine release syndrome. By figdraw.

Chimeric Antigen Receptor (CAR)-T cell technology is a cutting-edge immunotherapy approach designed to harness a patient’s own immune system by customizing autologous T-cells to express a CAR, which allows T cell to recognize and eliminate target cells bearing the cognate antigen. Costimulatory molecules and CAR immunoreceptors can further enhance targeted elimination of cells (**Figure 1**). Compared to antibody therapies, CAR-T cells act faster and in a more sustained fashion.

CAR-T cells have been engineered to target CD19 and other B-cell surface antigens with notable success in the treatment of relapsed or refractory B-cell cancers (1).

Inspired by the success in oncology, researchers are exploring the use of CAR approaches in autoimmune diseases, in which current treatments primarily rely on broad-spectrum immunosuppressive drugs for the suppression of self-reactive T cells and B cells that attack the host tissues causing inflammation and damage ultimately leading to loss of organ function.

The success of B-cell-targeted therapies and compelling data from preclinical models of systemic lupus erythematosus (SLE) (2) have supported the use of CAR-T cell therapy in the disease, and clinical trials have demonstrated that CD19 CAR-T cell therapy rapidly induced and sustained remission in refractory SLE without the adverse events typically associated with CAR-T therapy (3).

In addition to SLE, CAR-T cell therapy has also been shown to have considerable potential for type 1 diabetes (T1D), multiple sclerosis, autoimmune arthritis, graft-versus-host disease (GvHD), and host versus graft reaction (HvG). **Table 1** summarizes the ongoing clinical trials with CAR-T cell therapy in autoimmune diseases. These trials also include CAR-T cell therapy targeting CD19 and BCMA, the latter expressed by mature autoantibody-producing plasma cells resistant to immunosuppressive regimens.

**Table 1:** Registered clinical trials on CAR-T for Autoimmune Diseases on clinicalgov.com.

Target antigen/Drug	NCT Number	Conditions	Status	Number	Locations
**CD19, BCMA**	NCT06485232	Generalized Myasthenia Gravis, Multiple Sclerosis	Not yet recruiting	25	Xuanwu Hospital, Beijing
**IM19**	NCT06513429	Refractory SLE	Not yet recruiting	3	Peking University Third Hospital
**T cell**	NCT06352281	Immune Thrombocytopenia	Recruiting	10	920th Hospital of Joint Logistics Support Force of People’s Liberation Army of China
**CD19**	NCT06056921	Refractory Autoimmune Disease	Recruiting	24	Chongqing Precision Biotech Co.
**CD19**	NCT06106906	Active SLE	Recruiting	15	Wuhan Union Hospital, China
**LMY-920**	NCT06340750	Refractory SLE	Not yet recruiting	18	Luminary Therapeutics
**CD19-BCMA**	NCT06371040	Refractory Generalized Myasthenia Gravis	Not yet recruiting	12	Tang-Du Hospital
**BCMA/CD19**	NCT06428188	Relapsed/Refractory Autoimmune Disease	Recruiting	60	Essen Biotech
**CD19**	NCT06150651	SLE	Recruiting	6	Chulalongkorn University
**CD19-BCMA**	NCT06350110	SLE	Not yet recruiting	75	Essen Biotech
**CD19-CD3E**	NCT06373081	Relapsed/Refractory Autoimmune Disease	Recruiting	6	Shanghai Changzheng Hospital
**CD7**	NCT05239702	Crohn Disease, Ulcerative Colitis, Dermatomyositis, Still Disease	Recruiting	75	Zhejiang University
**CD19/BCMA**	NCT05263817	POEMS Syndrome, Amyloidosis, Autoimmune Hemolytic Anemia, Vasculitis	Recruiting	75	Zhejiang University
**BCMA**	NCT06277427	Refractory ANCA Associated Vasculitis, Lupus Nephritis	Recruiting	24	Tongji Hospital
**CD19-BAFF**	NCT06279923	Autoimmune Disease	Recruiting	45	Zhejiang University
**CD19/BCMA**	NCT05085444	Scleroderma	Recruiting	9	Zhejiang University
**CD19/BCMA**	NCT05085431	Sjögren’s Syndrome	Recruiting	9	Zhejiang University
**CD19/BCMA**	NCT05085418	Refractory Immune Nephritis	Recruiting	9	Zhejiang University
**CD19/BCMA**	NCT05030779	Refractory SLE	Unknown status	9	Zhejiang University
**CD19**	NCT06420154	Relapsed/Refractory Autoimmune Disease	Not yet recruiting	9	First Affiliated Hospital of Wenzhou Medical University
**BRL-301**	NCT05859997	Relapsed/Refractory Autoimmune Disease	Recruiting	15	Bioray Laboratories
**CD19**	NCT06138132	Multiple Sclerosis	Recruiting	12	Stanford University
**CD19**	NCT06342960	Lupus Nephritis	Recruiting	32	Kyverna Therapeutics
**CD19**	NCT05828225	Generalized Myasthenia Gravis	Recruiting	9	Zhejiang University
**CD19**	NCT05938725	Lupus Nephritis	Recruiting	32	Kyverna Therapeutics
**ATHENA**	NCT06373991	SLE	Not yet recruiting	12	EdiGene Inc.
**CD19**	NCT03030976	SLE	Unknown status	5	Shanghai GeneChem Co.
**CD19**	NCT06298019	Dermatomyositis	Not yet recruiting	21	Stanford University
**CD19**	NCT06451159	Progressive Multiple Sclerosis	Recruiting	10	University of California, San Francisco
**BRL-301**	NCT05988216	SLE	Recruiting	12	Bioray Laboratories
**CD19**	NCT06400303	Systemic Sclerosis	Not yet recruiting	21	Kyverna Therapeutics
**CD19**	NCT06347718	SLE, Systemic Sclerosis	Recruiting	24	University of Erlangen-Nürnberg Medical School
**CD19-BCMA**	NCT05858684	SLE	Recruiting	18	RenJi Hospital
**CD19**	NCT06212154	Autoimmune Hemolytic Anemia	Recruiting	13	Institute of Hematology & Blood Diseases Hospital, China
**CD19**	NCT06340490	SLE	Not yet recruiting	24	Guangdong Ruishun Biotech Co.
**CD20/BCMA**	NCT06249438	Relapsed/Refractory Autoimmune Disease	Recruiting	30	RenJi Hospital
**CD19**	NCT06384976	Systemic Sclerosis	Not yet recruiting	120	Kyverna Therapeutics
**BCMA**	NCT06519565	Recurrent/Refractory Immune Thrombocytopenia	Not yet recruiting	9	Wuhan Union Hospital, China
**CD19**	NCT06193889	Myasthenia Gravis	Not yet recruiting	20	Kyverna Therapeutics
**CD19/BCMA**	NCT06497361	Lupus Nephritis	Recruiting	30	Tongji Hospital
**BCMA**	NCT06497387	Lupus Nephritis, IgG4-related Disease	Recruiting	30	Tongji Hospital
**CT103A**	NCT04561557	Autoimmune Diseases of the Nervous System	Recruiting	36	Tongji Hospital
**MB-CART19.1**	NCT06189157	SLE	Not yet recruiting	29	Miltenyi Biomedicine GmbH
**CD19**	NCT06414135	Systemic Sclerosis	Recruiting	6	Renji Hospital
**CD19**	NCT06508346	ANCA Associated Vasculitis	Recruiting	12	The Children’s Hospital of Zhejiang University School of Medicine
**BCMA-CD19**	NCT05474885	SLE	Recruiting	15	iCell Gene Therapeutics
**CD19**	NCT06222853	SLE	Recruiting	19	The Children’s Hospital of Zhejiang University School of Medicine
**CD19**	NCT06429800	Lupus Nephritis	Not yet recruiting	26	Atara Biotherapeutics
**CD19**	NCT05459870	Autoimmune Diseases	Recruiting	30	Shenzhen Geno-Immune Medical Institute
**CD19, CD20**	NCT06153095	SLE	Recruiting	30	ImmPACT Bio
**CD19**	NCT06475495	Rheumatoid Arthritis	Not yet recruiting	13	Charite University, Berlin, Germany
**CD19**	NCT06294236	SLE, Anti-Neutrophil Cytoplasmic Antibody-Associated Vasculitis	Recruiting	36	Sana Biotechnology
**CD19**	NCT06316791	SLE	Recruiting	24	Juventas Cell Therapy Ltd.
**CD19/20**	NCT06462144	SLE, ANCA-Associated Vasculitis, Idiopathic Inflammatory Myopathy	Recruiting	36	The Affiliated Nanjing Drum Tower Hospital of Nanjing University Medical School
**BCMA, CD19**	NCT06435897	Autoimmune Diseases	Recruiting	30	Shenzhen Geno-Immune Medical Institute
**CD19**	NCT05765006	SLE	Recruiting	24	Shanghai Ming Ju Biotechnology Co.
**CD19**	NCT06361745	Autoimmune Diseases	Recruiting	10	PersonGen BioTherapeutics (Suzhou) Co.
**CD20**	NCT06375993	Autoimmune Diseases, Lupus Nephritis	Not yet recruiting	40	Adicet Therapeutics
**GC012**	NCT06419166	Refractory Generalized Myasthenia Gravis	Not yet recruiting	18	Zhejiang University
**FKC288**	NCT06285279	Autoimmune Diseases	Recruiting	24	Nanjing University School of Medicine
**BCMA**	NCT04146051	Generalized Myasthenia Gravis	Recruiting	30	Cartesian Therapeutics
**CD19**	NCT06417398	Autoimmune Diseases	Not yet recruiting	10	PersonGen BioTherapeutics (Suzhou) Co.
**CD19**	NCT05869955	Autoimmune Diseases	Recruiting	129	Juno Therapeutics, Inc., Bristol-Myers Squibb Company
**Relma-cel**	NCT06297408	SLE	Not yet recruiting	24	Shanghai Ming Ju Biotechnology Co.
**CD19**	NCT06333483	SLE	Recruiting	12	Autolus Limited
**CNCT19**	NCT05930314	SLE	ENROLLING BY INVITATION	12	Peking Union Medical College Hospital
**Descartes-08**	NCT06038474	SLE	Recruiting	30	Cartesian Therapeutics
**CD19**	NCT06328777	Systemic Sclerosis	Recruiting	12	Cabaletta Bio
**CD19**	NCT06465147	SLE	Not yet recruiting	12	Seattle Children’s Hospital
**CD19**	NCT06220201	Multiple Sclerosis	Recruiting	98	Juno Therapeutics, Inc., Bristol-Myers Squibb Company
**CD19**	NCT06152172	Autoimmune Diseases	Not yet recruiting	24	University of Pennsylvania
**YTB323**	NCT05798117	SLE, Lupus Nephritis	Recruiting	24	Novartis Pharmaceuticals
**BCMA, CD19**	NCT06503224	Autoimmune Diseases	Recruiting	18	The First Affiliated Hospital of University of Science and Technology of China
**CABA-201**	NCT06121297	SLE, Lupus Nephritis	Recruiting	12	Cabaletta Bio
**CABA-201**	NCT06359041	Generalized Myasthenia Gravis	Recruiting	12	Cabaletta Bio
**CABA-201**	NCT06154252	Idiopathic Inflammatory Myopathy	Recruiting	18	Cabaletta Bio
**MuSK-CAART**	NCT05451212	MuSK Myasthenia Gravis	Recruiting	24	Cabaletta Bio

Number: Number of patients enrolled or being enrolled.

Despite the promise, significant challenges hinder broader applications of CAR-T therapy in autoimmune diseases. Side effects of CAR-T therapy include cytokine release syndrome (CRS), neurotoxicity, and hematocytopenia (4). Additionally, the economic burden of CAR-T therapy is substantial, largely due to manufacturing expenses and the cost of pre-treatment for lymphocyte depletion and the management of possible adverse events, thus limiting access to these therapies (5).

**Figure 2:**
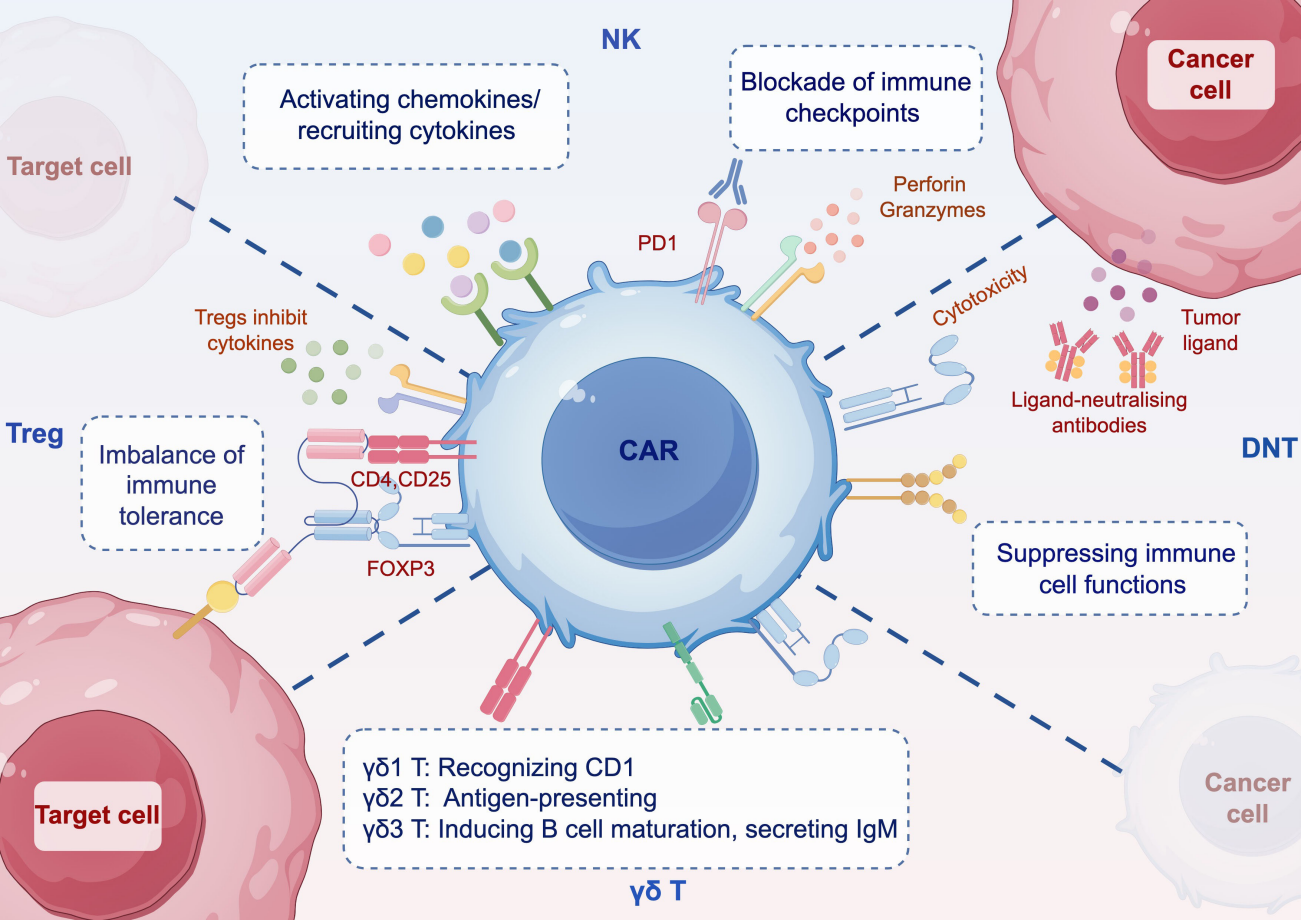
Schematic mechanisms of immune cells with CAR. DNT can inhibit the function of immune cells such as CD4^+^ and CD8^+^ T cells, B cells, dendritic cells, NK cells, etc. CAR-DNT cells also have potent antitumor properties. Tregs have immunosuppressive capacity and express CD4, CD25, and the master transcription factor FOXP3. CAR-Treg can modulate the imbalance of immune tolerance. γδ T cells are divided into three main classes *in vivo*, and targeting γδ T cells can activate or inhibit their functional responses. CAR-γδ T is unaffected by antigen processing and can directly kill tumor cells or target cells. CAR-NK cells can induce an early onset of antitumor activity. Blocking immune checkpoints, CAR-T cells are recruited through chemokines. CAR Chimeric antigen receptor, DNT double negative T cell, Treg regulatory T cell, NK nature killers. By figdraw.

More recently, CAR engineering of selected immune cells for immunotherapy against tumors and autoimmune diseases has broadened the focus to include double-negative T (DNT) cells, T regulatory cells (Tregs), γδ T cells, and natural killer (NK) cells (**Figure 2**). This review examines the development, current status, challenges and prospects of CAR-DNT cell, CAR-Tregs, CAR-γδ T cell and CAR-NK cell therapies for the treatment of autoimmune diseases, as well as their potential advantages and limitations as compared to the use of CAR-T cells.

## CAR-DNT

T cells express either αβ or γδ chain receptors, in addition to CD4 or CD8 (6). However, a specialized subpopulation of T cells characterized by the absent expression of CD4 and/or CD8, the CD3^+^CD4^-^CD8^-^ double-negative T (DNT) cells, accounts for approximately 1-5% of peripheral blood mononuclear cells (PBMCs), both in humans and in mice (7). Like all T cells, DNT cells express either the TCR αβ chain or the γδ chain, enabling them to recognize and respond to antigenic peptides in the adaptive immune response. The precise origin of human DNT cells remains uncertain, although it has been suggested that they may also derive from tissues and organs other than the thymus (8, 9). Functionally, DNT cells have inhibitory effects on CD4^+^ and CD8^+^ T cells, B cells, dendritic cells (DCs) and NK cells. This inhibitory capacity contributes to their suppressive role in GvHD and HvG reactions (10). Additionally, DNT cells have potent anti-tumor properties, reducing cancer cell proliferation and infiltration through mechanisms such as elevated production of interferon-γ (IFN-γ) and the expression of Fas ligand (FasL) (11, 12). Notably, DNTs are not restricted by the MHC and can be efficiently expanded *in vitro*, killing allogeneic and autologous leukemic cells through perforin- and granzyme-dependent processes (13–15).

In addition to their use in oncology, DNTs hold considerable potential for cellular immunotherapy. They can effectively inhibit CD4^+^ T cell proliferation and selectively suppress mammalian target of rapamycin (mTOR) signaling in these cells, altering metabolic patterns and functions to mimic long-lived central memory T cells and enhancing cell survival (16). Moreover, DNT cells can proliferate in inflamed tissues and contribute to the pathogenesis of autoimmune inflammatory diseases including SLE (17, 18), in which interleukin (IL)-17-producing DNT cells associate with progression of kidney disease, making them potential targets in lupus nephritis (19). Of interest, transfer of DNT cells into psoriatic mice associated with improvement of the inflammatory skin condition (20).

In 2000, Zhang et al. demonstrated antigen-specific inhibitory function of DNT cells in graft rejection in mice (21). In 2022, the same team successfully engineered DNT cells with an anti-CD19-CAR and assessed safety and efficacy in a mouse tumor xenograft model (22). The work confirmed the potential of CAR-DNT cells to target hematologic and solid tumors and reduce/delay acute GvHD (23). Recent studies have assessed the safety and anti-tumor effectiveness of a novel CAR-DNT cell therapy, RJMty19, in patients with relapsed/refractory large B-cell lymphoma (LBCL). This first human, open-label, single-dose, phase 1 study indicated that CD19-CAR-DNT cells are well-tolerated in patients with LBCL and show robust anti-tumor activity. Importantly, neither severe CRS or neurotoxicity was observed, nor were any cases of GvHD or dose-limiting toxicities (DLTs) reported (24). These findings suggest that CAR-DNT therapy may offer a better safety profile compared to conventional CAR-T cell therapies. Furthermore, DNT cells expanded from healthy volunteers under good manufacturing practice (GMP) conditions can be cryopreserved, maintaining their functionality over time both *in vitro* and *in vivo*.

The advancement in cellular formulation—from fresh to cryopreserved—holds significant potential, positioning cellular therapies to become readily available as traditional pharmaceuticals. While further investigations into the specific roles and mechanisms of DNT cells in inflammatory or tumor environments are needed, there are currently four CAR-DNT studies registered on Clinicaltrials.gov, two of which focusing on safety and efficacy of CD19-CAR-DNT in the treatment of autoimmune diseases (**Table 2**).

**Table 2:** Registered clinical trials on CAR-DNT on clinicalgov.com.

Target antigen/Drug	NCT Number	Conditions	Status	Number	Locations
**CD19**	NCT06316076	SLE, Anti-Neutrophil Cytoplasmic Antibody-Associated Vasculitis, Idiopathic Inflammatory Myopathies, Systemic Sclerosis	Recruiting	48	Ren Ji Hospital, Shanghai JiaoTong University
**RJMty19**	NCT06340490	SLE	Not yet recruiting	24	Peking University People’s Hospital, Nanjing Drum Tower Hospital, Renji Hospital, Changhai Hospital
**CD19**	NCT05453669	B-cell non-Hodgkin’s Lymphoma	Recruiting	12	2nd Affiliated Hospital, Zhejiang University
**RJMty19**	NCT06314828	B-cell non-Hodgkin’s Lymphoma	Not yet recruiting	30	Guangdong Ruishun Biotech Co., Ruijin Hospital, Southern Medical University, Beijing GoBroad Hospital

Number: Number of patients enrolled or being enrolled.

## CAR-Tregs

Tregs are a subset of T cells known for their immunosuppressive capabilities and express CD4, CD25, and the master transcription factor FOXP3. They can be classified into natural Tregs (nTregs), which develop in the thymus, and peripherally induced Tregs (pTregs), the latter differentiating from peripheral naïve CD4^+^CD25^-^ T cells in the presence of tolerogenic cytokines (25). Since Tregs inhibit T effector cells, in the tumor microenvironment they contribute to cancer immune evasion and represent therefore a major barrier to cancer immunotherapy. On the contrary, a defective Treg number and/function promotes T effector responses to self-antigens and autoimmune disease (26, 27).

Several studies have shown that Tregs are promising for treating autoimmune diseases and preventing GvHD. In 2009, *in vitro*-expanded autologous polyclonal Tregs were successfully transferred to patients with acute or chronic GvHD (28). In autoimmune diseases, antigen-specific Tregs show higher immunosuppressive potency in various preclinical murine models as compared to classical polyclonal Tregs (29–31). Importantly, antigen specificity of the Tregs can achieved by engineering them to express transgenic TCRs or CARs. These Tregs predominantly localize to sites where target antigens are expressed, reducing risks of systemic immunosuppression. While TCR-engineered Tregs are MHC-dependent, CAR Tregs are MHC-independent and less reliant on IL-2 (32). CARs can also recognize non-protein targets like carbohydrate and glycolipid molecules (33), and CAR Tregs show considerable potential for various immune disorders. For example, CD19-CAR Tregs inhibit antibody production and B cell differentiation, effectively suppressing B cell proliferation and thus a key immune activity implicated in the pathogenesis of SLE. In murine models of SLE, a single infusion of FOXP3-overexpressing anti-CD19 CAR Tregs limited autoantibody production, reduced lymphopenia, and helped restore immune cell compartments in lymphoid organs without toxicity (34). Another study showed that injecting immunodeficient mice reconstituted with human PBMCs with CD19-CAR Tregs suppressed antibody production and reduced GvHD risk (35).

Importantly, in SLE patients, CAR-Tregs efficiently restored immune homeostasis without adverse side effects (3, 34, 35).

The importance of antigen-specific stimulation for CAR-Tregs in CEA transgenic mice with colitis also showed potential in ameliorating ulcerative colitis and hindering colorectal cancer progression (36). Another study designed CAR-Tregs specific for myelin oligodendrocyte glycoprotein to inhibit experimental autoimmune encephalomyelitis (37), a model of multiple sclerosis (MS). In this model, co-expression of FOXP3 and CAR promoted Treg differentiation from naïve CD4 T cells, and the engineered Tregs suppressed ongoing encephalomyelitis (38). Also CAR-Tregs for type 1 diabetes (T1D) localized to the pancreas and regulated localized cellular damage (39).

So far, all phase I/II clinical studies have demonstrated safety of CAR-Treg immunotherapy (40), with no added risk of infection or cancer. Similar to clinical trials on polyclonal Tregs (41), CAR-Tregs show limited persistence. Adjusting treatment regimens, such as combining CAR-Tregs with low-dose IL-2 or removing lymphocytes beforehand might improve viability and persistence (42).

## CAR-γδ T

γδ T cells represent a small fraction of T cells, about 1-5% of PBMC. Unlike traditional CD4^+^ and CD8^+^ αβ T cells, γδ T cells have innate features such as MHC-independent antigen recognition similar to NK cells and cytotoxic activity. Additionally, they produce chemokines, cytokines, and inflammatory and cytotoxic mediators that enhance inflammatory responses and regulate the differentiation and apoptosis of damaged cells, forming a first line of defense against tumors and infection (43). Human γδ T cells are categorized into three main types based on their δ-chain expression: Vδ1, Vδ2, and Vδ3 T cells. The majority (50-90%) of γδ T cells in the human blood express the Vδ2 TCR. Activated Vδ2 T cells function as professional antigen-presenting cells (APCs), presenting antigens and expressing co-stimulatory molecules and adhesion molecules such as MHC II, CD80, and CD86 (44). The αβ TCRs are restricted to the recognitions of antigen-derived peptides presented by MHC/human leukocyte antigens (HLA) molecules, thus distinguishing them from γδ T cells-which are not constrained by antigen processing and MHC/HLA restriction (45).

In preclinical studies, CAR-γδ T cells showed antitumor efficacy in leukemia models. Transduction of polyclonal γδ T cells with CD19-targeted CAR enhanced IFN-γ and tumor necrosis factor (TNF)-α responses and significantly reduced leukemic load (with comparable transduction efficacy and cytotoxicity to standard CAR-T *in vivo*) (46, 47). However, for the aggressive ALL cell line in mice, it was impossible to completely clear it, because of the limited persistence of CAR-γδ T cells. Yet, repeated infusions of CAR-γδ T cells improved the anti-leukemic effects (47).

For solid tumors, CAR-γδ T cells may act as professional APCs, inducing endogenous immunity in response to antigenic heterogeneity (48).

In autoimmune diseases, specifically in rheumatoid arthritis, γδ T cells contribute to inflammation by secreting cytokines and inducing inflammatory cells. Several studies have shown that γδ T cells strongly regulate Th17 autoimmune responses in experimental autoimmune uveitis (49) and interact with DCs (50). Higher numbers of γδ T cells have also been observed in the MS white matter plaques and cerebrospinal fluid as compared to peripheral blood (51). An increase in circulating γδ T cells can also be seen in relapsing-remitting MS (RRMS) patients (52). However, numbers of total γδ and γδ2 T cells were significantly lower in MS patients compared to healthy controls, possibly due to apoptosis or migration of γδ2 T cells to the central nervous system (CNS) (53).

γδ T cells help control inflammation and promote disease repair in the CNS through Fas/FasL-induced apoptosis of encephalitogenic T cells. Timely resolution of inflammation is crucial to prevent irreversible CNS damage in chronic diseases (54). Thus, the clinical potential of CAR-modified γδ T cell is enormous, also because CAR-γδ T cells contribute to humoral immunity by targeting and promoting B cell maturation, antibody production, and class switching. They also indirectly regulate αβ T cell activity by activating NK cells, DCs, and B cells. Although CAR γδ T cell immunotherapy is still at early stages, it has shown promise in the field of oncology as well as autoimmune diseases. A total of eight studies on CAR-γδ T have been registered on Clinicaltrials.gov, including two on autoimmune diseases, one for refractory/moderately severe SLE and one for lupus nephritis (**Table 3**).

**Table 3:** Registered clinical trials on CAR-γδT on clinicalgov.com.

Target antigen/Drug	NCT Number	Conditions	Status	Number	Locations
**CD19**	NCT06106893	Refractory/Moderate-to-severe SLE	Recruiting	15	Wuhan Union Hospital, Guangzhou Bio-gene Technology Co., Melbourne
**CD20**	NCT06375993	Lupus Nephritis	Not yet recruiting	40	Adicet Therapeutics

Number: Number of patients enrolled or being enrolled.

## CAR-NK

NK cells are innate immune cells that act as the first line of defense against tumors or virally infected cells independently of MHC molecules. NK cell function is modulated by a complex array of activating and inhibitory receptors that determines whether NK cells initiate killing activity against abnormal cells or maintain tolerance to healthy cells (55). At least 90% of peripheral blood NK cells are CD56^dim^CD16^bright^, representing the ultimate stage of NK cell maturation. Conversely, CD56^bright^ NK cells are immature populations that more closely resemble helper cells and secrete large amounts of cytokines such as IFN-γ, TNF-β, and granulocyte-macrophage colony-stimulating factor (56, 57). CAR significantly enhances the specificity and potency of NK cells. Due to their unique features, CAR NK cells possess both CAR-mediated targeted killing ability and inherent anti-tumor properties. They can recognize and kill tumor cells even when CAR targets are dowregulated or absent, reducing the chances that tumor cells escape detection, thereby increasing therapeutic efficacy (58). Additionally, NK cells have a lower risk of inducing GvHD (59) and are less likely to cause CRS and severe neurotoxicity than activated T cells (60). Interestingly, NK cells have a limited lifespan, averaging about two weeks (61). This means that any potential toxicity from CAR-NK cells can be self-limiting as the cells naturally disappear. However, this also implies that repeated infusions of CAR-NK cells might be necessary to maintain remission. In 2020, Liu et al. published a phase I clinical trial of CAR-NK cells against CD19-positive lymphoid tumors (62). In this trial, 11 patients with B-lymphoid malignancies were treated, of which 7 achieved complete remission, 6 tested negatives for minimal residual disease, and none experienced increased inflammatory cytokines or adverse effects such as CRS, neurotoxicity, hemophagocytic lymphohistiocytosis, or GvHD. In another phase I trial, NK-92-derived CD33-CAR-NK cells showed good tolerability in three patients with acute myeloid leukemia (NCT02944162). These results demonstrate that CAR-NK cells are a safe strategy for cancer treatment. In autoimmune diseases, Meng et al. developed a chimeric autoantibody receptor (CAAR) targeting the autoantigen La/SSB (63), which is associated with several autoimmune diseases. They introduced this CAAR into NK92MI cells, enabling them to selectively target and destroy La/SSB-reactive autoreactive B-cell clones, providing a new approach to autoimmune disease therapy. Additionally, King et al. showed that treating SLE mice with CAR NK-92 cells reduced splenomegaly and the numbers of CD4^+^ T cells (64). While CAR NK therapy for autoimmune diseases shows promise, more clinical studies are needed to support and explore its potential. Considering the lifespan of mature NK cells and their transient nature *in vivo*, persistence will be a major issue affecting clinical outcomes. Of note, it remains controversial whether CAR-NK cell transfusion requires the same lymphocyte-clearing chemotherapy with fludarabine and cyclophosphamide as CAR-T therapy. To date, all published clinical trials have administered lymphocyte-clearing chemotherapy prior to CAR-NK treatment, which may be due to the fact the enrolled patients with refractory disease had received other targeted therapies, such as CAR-T therapy, prior to CAR-NK (65). Alternatively, the investigators administered uniform lymphatic clearance prior to clinical trial in order to observe the effects of different doses of CAR-NK on patients (66). In all, whether eliminating lymphatic chemotherapy is imperative during CAR-NK cell therapy still needs to be explored. Currently, there are 104 CAR-NK programs registered on Clinicaltrials.gov, with 10 focused on autoimmune diseases (**Table 4**).

**Table 4:** Registered clinical trials on CAR-NK on clinicalgov.com.

Target antigen/Drug	NCT Number	Conditions	Status	Number	Locations
**CD19**	NCT06318533	Autoimmune Diseases	Recruiting	15	Affiliated Hospital of Jiangsu University
**F01**	NCT06208280	Autoimmune Diseases	Recruiting	20	Ren Ji Hospital South Campus, JiaoTong University
**CD19**	NCT06010472	SLE	Recruiting	12	Changhai Hospital, Shanghai
**CD19**	NCT06337474	Thrombocytopenia Alloimmune	Not yet recruiting	9	Changzhou No. 2 People’s Hospital, Rui Therapeutics Co, Seattle
**CNTY-101**	NCT06255028	Refractory/Moderate-to-severe SLE	Not yet recruiting	26	Century Therapeutics, Inc, Philadelphia.
**CD19**	NCT06421701	Refractory/Relapsed SLE	Not yet recruiting	10	Second Affiliated Hospital, Zhejiang University
**CD19**	NCT06464679	Autoimmune Diseases	Recruiting	72	Changhai Hospital
**CD19**	NCT06518668	SLE	Recruiting	6	Columbia University
**F01**	NCT06468683	SLE	Not yet recruiting	50	Shanghai Simnova Biotechnology Co.
**CD19**	NCT06377228	Refractory Lupus Nephritis	Not yet recruiting	20	Takeda

Number: Number of patients enrolled or being enrolled.

## Discussion

CAR-T cell therapy has been a landmark achievement in oncology yet still faces obstacles including the immunosuppressive and immunoexclusive tumor microenvironment, tumor antigen heterogeneity, and poor T cell trafficking. For autoimmune diseases, CAR therapy is still in its infancy. Although only a few autoimmune diseases may be suitable for CAR cell therapy, the large number of patients affected by autoimmune diseases means that as the number of approved clinical therapies increases, so will the total number of patients eligible for CAR cell therapy.

Importantly, combinations of CAR immune cells supporting conventional CAR-T cells are overcoming current challenges in oncology treatment. For example, CAR-DNT are not restricted by MHC and efficiently expand *in vitro*; CAR-Tregs act as naturally inhibitory; and CAR-γδ T cells act as APCs, cross-presenting antigens to conventional αβ CAR-T and endogenous T cells. Similar to CAR-γδ T cells, CAR-NK cells can be used in combination with conventional CAR-T cells or as a bridging therapy prior to CAR-T therapy. Therefore, combining various types of CAR cells to provide synergistic effects is a promising direction for future research.

While some CAR-T products for treating lymphomas and leukamia have been approved by the FDA and have shown impressive anti-tumor effects, CAR-T therapies remain expensive. Cells derived from induced pluripotent stem cells(iPSCs) induced by genetic factors or chemical reagents provide a solution for constructing large-scale standardized CAR cells that can significantly reduce costs. However, inducing functional, modulable immune cells from iPSCs remains a technical challenge.

Overall, despite the many challenges in the development of CAR cell therapy, next generations of CAR beyond CAR-T are emerging with improvement on manufacturing and storage process of CARs, more targeted design, applications in solid tumors, and reduced side effects including CRS, neurotoxicity and thrombocytopenia. As CAR will target diverse immune cells, durable disease remission and more safety will improve prospects of therapeutic management of tumors and autoimmune diseases.
